# Are Reusable Dry Electrodes an Alternative to Gelled Electrodes for Canine Surface Electromyography?

**DOI:** 10.3390/ani15202959

**Published:** 2025-10-13

**Authors:** Ana M. Ribeiro, I. Brás, L. Caldeira, J. Caldeira, C. Peham, H. Plácido da Silva, João F. Requicha

**Affiliations:** 1 Department of Veterinary Sciences, University of Trás-os-Montes e Alto Douro (UTAD), 5000-801 Vila Real, Portugal; 2 Animal and Veterinary Research Centre (CECAV), UTAD, Associate Laboratory for Animal and Veterinary Sciences (AL4AnimalS), 5000-801 Vila Real, Portugal; 3 Egas Moniz Center for Interdisciplinary Research (CiiEM), Egas Moniz School of Health & Science, 2829-511 Caparica, Portugal; 4 Department of Bioengineering, Instituto Superior Técnico, 1049-001 Lisbon, Portugal; 5 Movement Science Group, Clinical Centre for Equine Health and Research, Clinical Department for Small Animals and Horses, University of Veterinary Medicine, 1210 Vienna, Austria; 6 Instituto de Telecomunicações, Universidade de Aveiro, 1049-001 Lisbon, Portugal

**Keywords:** surface electromyography, dry electrodes, canine, muscle activity

## Abstract

Despite its increasing use in veterinary rehabilitation, practical constraints—such as skin preparation and single-use electrodes—limit the wider adoption of surface electromyography (sEMG). Having conventional pre-gelled Ag/AgCl electrodes as reference, we made a pioneering comparison of the performance of reusable soft polymeric dry electrodes for recording paraspinal muscle activity in dogs during treadmill walking. Twelve clinically healthy Dachshunds from both genders were evaluated under two conditions, namely: (i) dry electrodes on untrimmed hair; and (ii) pre-gelled electrodes after trichotomy. Signals were acquired from the longissimus dorsi muscle at 1 kHz, processed with standardized filtering and rectification, and analyzed in both time and frequency domains. Dry electrodes yielded higher amplitude and Root Mean Square (RMS) values, but slightly lower power spectral density metrics when compared to pre-gelled electrodes. Nevertheless, frequency-domain results were broadly comparable between configurations. Dry electrodes reduce the preparation time, avoid hair clipping, and allow reusability without major signal degradation. While pre-gelled electrodes may still offer marginally superior stability during movement, our results suggest that soft polymeric dry electrodes present a feasible, less invasive, and more sustainable alternative for canine sEMG. These findings support further validation of dry electrodes in clinical populations, particularly for neuromuscular assessment in intervertebral disk disease.

## Simple Summary

Non-invasive assessment of muscle activity in veterinary patients can be highly advantageous, especially when dealing with neuromuscular disorders. A primary test for such a purpose is electromyography (EMG), particularly surface EMG (sEMG); however, its use in small animals is limited by the need for skin preparation and disposable electrodes. To our best knowledge, with this study, we evaluated for the first time whether reusable dry electrodes constitute a feasible alternative to conventional gel-based electrodes, through signals collected from 12 dogs during dynamic treadmill walking. Our findings show that, even without hair clipping, dry electrodes yield reliable frequency-domain data and higher amplitude readings comparatively to gel-based electrodes, offering a practical, faster, and more sustainable alternative for clinical and research use. Our results pave the way for a more widespread use of sEMG with dry electrodes in canine rehabilitation, e.g., for conditions such as intervertebral disk disease.

## 1. Introduction

Surface electromyography (sEMG) is a non-invasive diagnostic tool that records the electrical activity of muscles [1,2,3,4,5]. Measuring electrical signals generated by motor units’ action and muscle fiber recruitment offers meaningful insights into the mechanisms underlying muscle contraction and neuromuscular control [6]. sEMG has proven to be a valuable technique for assessing neuromuscular disorders, monitoring treatment progress, and evaluating therapeutic interventions, allowing clinicians to evaluate muscle function in dynamic activities; it is used in various disciplines, including basic research, clinical diagnostics, sports medicine, and rehabilitation [6,7,8,9,10,11]. Studies by Balbinot et al. 2021 and Mitchell et al. 2015 suggest that sEMG can be a useful tool in conjunction with current standard protocols (International Standards for Neurological Classification of Spinal Cord Injury—ISNCSCI, American Spinal Cord Association Impairment Scale—AIS and Graded Redefined Assessment of strength, sensibility and Prehension—GRASSP) for clinical assessment of individuals with spinal cord injury (SCI) [12,13]. sEMG amplitude has been associated with a measure of muscle vitality, is a reliable indicator of muscle recovery, and can identify residual muscle activity in patients with no observable movement below a spinal lesion [13]. Although, to the best of our knowledge, it is still unexplored in applications, beyond clinical assessment, EMG opens new avenues for the control of robotic devices, prosthetics, and exoskeletons in human healthcare [5,6,14,15].

In the context of veterinary rehabilitation, sEMG has emerged as a promising tool for the non-invasive measurement of muscular function in conscious patients, used increasingly in research of both equine and companion animal medicine [16,17,18].

Our study focuses on using sEMG in veterinary medicine, motivated by the lack of non-invasive and objective tools to assess neuromuscular function in animals with spinal cord disorders such as intervertebral disk disease (IVDD) undergoing rehabilitation medicine treatments. To our best knowledge, this study provides the first direct comparison of soft polymeric dry electrodes and gel-based electrodes for canine dynamic sEMG, addressing a key barrier to wider adoption of objective neuromuscular assessment tools in veterinary rehabilitation. With the use of sEMG, clinicians can develop targeted rehabilitation strategies [6] tailored to the individual needs of each patient, based on their neurological status and tolerance [19].

According to the Consensus for Experimental Design in Electromyography (CEDE)—an international initiative aimed at guiding best practices for the recording, analysis, and interpretation of EMG data [9,10,20,21], bipolar surface electromyography (sEMG) involves the differential amplification of signals detected by pairs of surface electrodes; the bipolar configuration is particularly advantageous, as it can better mitigate the effects of common mode noise. This technique is widely used for assessing superficial and easily accessible muscles, with the estimated activation level representing the activity of a relatively large number of muscle fibers. Furthermore, CEDE highlights that, as the interface between the tissue and the recording system, the selection of the appropriate EMG electrodes is of utmost importance.

In human sEMG, conventional silver/silver chloride (Ag/AgCl) electrodes are typically placed on the skin overlaying the target muscle [9]. However, in veterinary applications, sEMG presents additional challenges, including the need for skin preparation (i.e., trichotomy), to ensure adequate electrode adhesion and better manage behavioral constraints during data collection [16,17,18]. Most of the previous literature on animal sEMG primarily focuses on kinematics and locomotion function on muscles of horses and dogs, specifically limb muscles [22,23,24,25,26,27]; there is a gap in what concerns the evaluation of neuromuscular diseases. As such, our main objective was to evaluate the practical efficiency of sEMG in the paraspinal muscle region, using a completely different set of electrodes.

Particularly, intervertebral disk disease (IVDD) is a common condition in dogs that, among others, can lead to several serious myelopathies such as intervertebral disk herniation (IVDH), degenerative lumbosacral stenosis, or cervical spondylomyelopathy [28], which in turn can significantly impair quality of life and life expectancy. In fact, IVDD underlies the most common forms of intervertebral disk herniation (IVDH) [29], and it is estimated to account for approximately 21% of all neurological cases reported in domestic dogs. The overall incidence of this pathology across all domestic canine breeds is estimated at 2% to 3.5% [30]. A previous pilot study developed by Ribeiro et al. 2024 [22] involving IVDD patients and healthy controls, suggested a decrease in sEMG amplitude and an increase in sEMG frequency in exercises featuring more dynamic muscle activation in the group of animals affected with IVDD. This further reinforces that sEMG can be an added value tool to assess neuromuscular function, as it has the potential to categorize EMG signals between healthy animals and the ones affected with IVDD. Also, a study by Schwartz et al. 2024 [31], using sEMG in dogs that had undergone hemilaminectomy, concluded that post-hemilaminectomy dogs had greater hind limb activation when compared with normal dogs. Their results support the idea that sEMG can be valuable to evaluate muscle activity in dogs recovering from myelopathies/spinal decompression surgery.

Still, the broader use of sEMG in small animals has been limited by trichotomy, signal instability, and the need for specialized training in signal analysis [16,17,18]. Our study seeks to analyze the feasibility of soft polymeric dry electrodes for sEMG applications, comparatively to conventional gel-based electrodes in canine subjects. If successful, this procedure would greatly simplify the clinical use of sEMG in this context.

In general, soft polymeric dry electrodes produced higher amplitude and a larger root mean square error (RMSE). Regarding power spectral density (PSD) values, both dry and pre-gelled electrodes were similar, although the pre-gelled electrodes tended to have slightly higher spectral power. Our results indicate that dry electrodes offer practical advantages in terms of easier use and reduced preparation time, without compromising signal integrity, but pre-gelled electrodes remain advisable whenever possible.

## 2. Materials and Methods

### 2.1. Pre-Study Evaluation

The animal study protocol was approved by the Ethics Committee of Trás-os-Montes e Alto Douro University (Doc106-CE-UTAD-2024) for studies involving animals.

Before participating in any of the experimental procedures, all studied dogs underwent a clinical pre-assessment conducted by a Certified Canine Rehabilitation Veterinarian (*Canine Certified Canine Practitioner—CCRP*). This evaluation served not only to ensure that each animal was clinically fit to perform the planned tasks without discomfort or risk of injury, but also to confirm the absence of musculoskeletal or neurological conditions that could compromise the validity of the results. This pre-assessment consisted of a general health evaluation and a thorough orthopedic and neurological assessment [32,33,34].

### 2.2. Animal Selection

To isolate breed as a variable, only Dachshunds were selected. The inclusion of this breed in this study was based on two principal criteria: (i) its increasing popularity as a companion animal [35,36,37]; and (ii) its well-documented genetic predisposition to a broad spectrum of orthopedic and neurological disorders, with particular emphasis on IVDD which is estimated to affect 19–31% of these dogs during their lifetime, with the highest incidence occurring between 4 and 6 years of age [38,39,40,41]. This breed-specific anatomical and physiological predisposition frequently results in significant neuromuscular impairment, necessitating extended therapeutic interventions and an intensive requirement for physiotherapeutic and functional rehabilitation [42].

The main characteristics of the dogs recruited are summarized in Table 1.

From the 13 dogs initially recruited, one dog was excluded after clinical evaluation due to the presence of pain at vertebral column palpation. From the 12 dogs included in the study, four were females, three of which had ovariohysterectomy, and one was intact; the remaining eight were males, of which two were neutered and six were intact. The mean age of the animals was 3 ± 1.7 years (median of 3 years). The average body condition score was 5 ± 1 based on a 9-point scale, with individual scores ranging from 4/9 to 7/9.

Inclusion criteria required that animals had no history of lameness or orthopedic issues and exhibited normal findings on both orthopedic and neurological examinations. Furthermore, no animals under the age of one year or older than 7 years were included, as this could compromise the sEMG readings [43,44].

### 2.3. Clinical Evaluation

For the participants included in the study, a more comprehensive assessment was carried out in eight stages [32,33,34]:

- Collection of medical history;
- General health inspection;
- Standing inspection to assess muscle asymmetry, musculoskeletal deformities, and even weight distribution across limbs;
- Gait inspection with special attention to signs of lameness;
- Palpation of limb joints to confirm absence of crepitus, edoema, instability, or pain;
- Palpation of the spine and surrounding musculature, including careful manipulation of the cervical spine through its full range of motion (ROM) to ensure absence of pain;
- Proprioceptive testing and spinal reflexes on all four limbs;
- Specific orthopedic tests, such as the drawer test and tibial thrust.

### 2.4. Data Acquisition Setup

**Figure 1:**
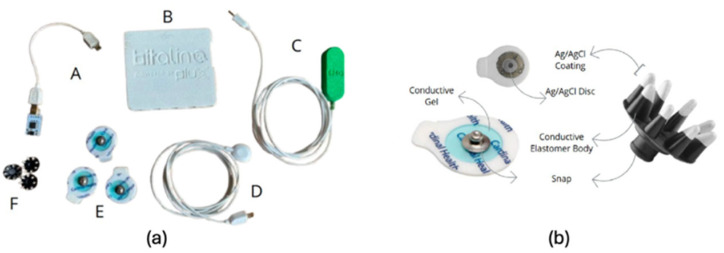
Surface Electromyography Setup. (**a**) Hardware devices used in the experimental setup, showing: A—Accelerometer (ACC); B—BITalino (r)evolution Core BT board; C—sEMG sensor; and D—1-lead electrode cable; E—Pre-Gelled Ag/AgCl electrodes; F—SoftPulse Flex dry electrodes (**b**) Electrodes used for signal acquisition: Pre-Gelled Ag/AgCl (left) and SoftPulse Flex dry (right).

The experimental setup was composed of a BITalino (r)evolution Core BT board (PLUX^®^, Lisbon, Portugal), one BITalino assembled sEMG sensor (PLUX^®^, Lisbon, Portugal), and a 1-lead electrode cable (for the reference electrode). Both pre-gelled Ag/AgCl (Kendall™H124SG, Leeds, UK) surface electrodes and soft polymeric dry electrodes (SoftPulse^TM^ Flex, Datwyler^®^, Altdorf, Switzerland) were used for signal acquisition (Figure 1). Specific characteristics of both electrode types and applications are described in Table 2. A BITalino accelerometer (ACC) sensor (PLUX^®^, Lisbon, Portugal) was also integrated into the setup and physically attached to the BITalino unit, which was secured to a harness worn by the dog. The accelerometer was used to monitor the animal’s movement and to assist in identifying stable signal segments in the subsequent sEMG processing stages. Data collection and visualization were performed using a laptop running the OpenSignals v2.2.5 (r)evolution software (PLUX^®^, Lisbon, Portugal).

**Figure 2:**
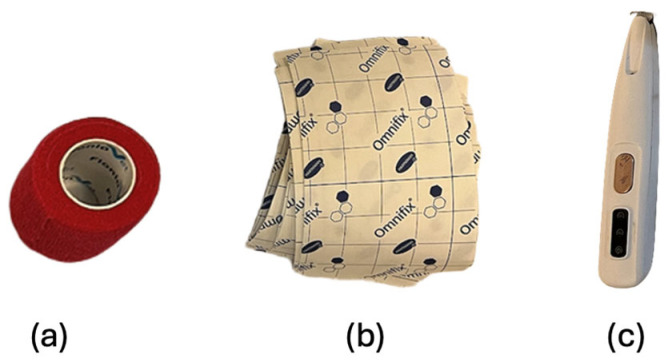
Additional material used in the study. (**a**) Co-adhesive flexible bandage 10 × 4.5 cm (Vet-flex, Kruuse^®^, Denmark). (**b**) Omnifix 10 × 10 cm elastic adhesive (Hartmann^®^, Germany). (**c**) Professional trimmer (IPX5 waterproof, China).

For sEMG device and electrode fixation purposes co-adhesive flexible bandage 10 × 4.5 cm (Vet-flex, Kruuse^®^, Langeskov, Denmark) was used, as well as Omnifix 10 × 10 cm elastic adhesive (Hartmann^®^, Heindenheim, Germany) (Figure 2). Lastly, when trichotomy was necessary, a professional trimmer (IPX5 waterproof, Yiwu, China) for small areas was used, to minimize the amount of hair clipped, the trimmer chosen had a blade of proximally 20 mm width and a rounded R-type design that is believed to prevent skin scratching (Figure 2).

### 2.5. Skin Preparation and Exercise

Measurements from all 12 participants were performed twice, first with the soft polymeric dry electrodes and untrimmed hair conditions, and afterwards with the pre-gelled Ag/Ag electrodes and trimmed hair conditions. Electrodes were placed on the same anatomical location, overlaying the *longissimus dorsi* muscle between thoracic vertebra 12 (T12) and lumbar vertebra 2 (L2), 0.5 cm lateral to the spinous processes on the right side of the body, and on the same day. In both evaluations, the reference electrode (grounding)—Ag/Ag electrodes were used—was positioned on a bony prominence—medial aspect of the tarsus (medial tibial malleolus)—to minimize interference and provide a stable baseline. These electrodes were secured using a co-adhesive flexible bandage. Before positioning, trichotomy was performed, and skin was cleaned using 70% alcohol wipes.

**Figure 3:**
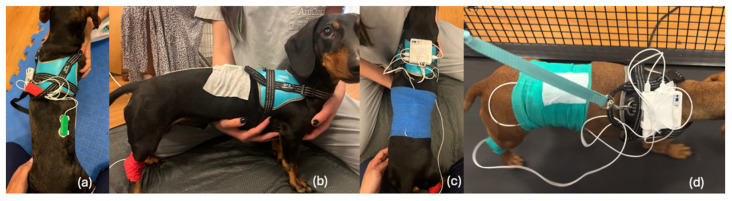
Experimental setup according to a previously published method [22], applied to four different subjects: (**a**) Placement electrodes overlaying the longissimus dorsi muscle between thoracic vertebra 12 (T12) and lumbar vertebra 2 (L2); (**b**) reference electrode (grounding)—positioned on a the medial aspect of the tarsus, secured using co-adhesive flexible bandage (red), and vertebral electrodes secured with Omnifix elastic adhesive (white); (**c**) sEMG device positioned in the participants harness and vertebral electrodes secured with a second layer using co-adhesive flexible bandage (blue); (**d**) participant walking on professional veterinary treadmill. The white bandage shown in the image of subject (**d**) was placed solely for visualization purposes (to illustrate the movement during adhesive bandage application) and is not intended to secure the electrodes.

In the first stage (untrimmed hair condition), two dry electrodes were applied to the longissimus dorsi muscle (Figure 3), ensuring firm contact with the skin and proper parallel alignment with the muscle fibers. To guarantee adequate adhesion and reduce skin impedance, before electrode placement, the skin was cleaned using 70% alcohol wipes. After placement, electrodes were secured in place with Omnifix elastic adhesive and, to guarantee minimal displacement during walking, 10 × 4.5 cm co-adhesive flexible bandage was also applied. To ensure consistency, whenever the electrode setup shifted or fell out of place during signal acquisition, the preparation process was restarted entirely from the beginning. This was performed to maintain uniformity and avoid introducing artifacts or inconsistencies in the data. Our goal was to minimize the effects of movement as much as possible.

Each participant was placed on a professional veterinary treadmill (FitFurLife^®^ Professional Treadmill, Surrey, UK) and encouraged to walk at a constant, comfortable speed of 1.93 km/h to promote regular and consistent muscle activation of the spinal muscles. To ensure stable gait cycles and minimize motion artifacts, each dog was allowed to walk on the treadmill for a few seconds until a steady and repeatable walking pattern was achieved. Once a regular gait was confirmed, signal acquisition was performed in two stages. First, data was collected while the dog walked steadily on the treadmill for approximately 60 s. After this recording, the experiment proceeded to the second stage (trimmed condition), in which the hair in the electrode placement area was carefully trimmed (trichotomy), maintaining the same position and alignment of the electrodes relative to the muscle fibers.

The location for trichotomy was guided by the faint imprint left by the dry electrodes on the fur, which enabled precise trimming in the same spot used during the untrimmed condition; this ensured consistency in electrode positioning across both stages of the experiment. The skin was again cleaned with alcohol wipes, and pre-gelled Ag/AgCl electrodes were applied using the exact same technique previously used; the ground treadmill exercise was then performed under the same conditions and for the same duration to allow a direct comparison between electrode types.

An accelerometer was used simultaneously and synchronously recorded with the sEMG sensor to monitor the dog’s movement, enabling the identification of stable gait cycles and signal segments suitable for amplitude and spectral analysis. Signals were also acquired at 1 kHz using the BITalino system and OpenSignals software (Section 2.3), with sessions labeled by electrode type and hair condition for analysis and date (e.g., SH3/flex/notricho/07-05 vs. SH3/Ag/AgCl/tricho/07-05).

### 2.6. Signal Processing

All raw sEMG signals acquired in both settings were processed using the same pipeline to ensure consistency and comparability across electrode types and hair conditions. Each raw signal was first filtered using a 6th-order digital band-pass filter with a passing band between 25 and 480 Hz to isolate the relevant muscle activity and remove unwanted noise; this specific frequency range is chosen because it encompasses the typical frequencies produced by muscle contractions, while simultaneously attenuating low-frequency motion artifacts and high-frequency noise [45,46].

Additionally, a notch filter centered at 50 Hz with a quality factor of 30 (which indicates the filter’s sharpness in attenuating this specific frequency) was applied to suppress powerline interference, i.e., unwanted electrical noise at the frequency of the power grid in Portugal [47]. When cardiac interference (i.e., electrocardiography contamination) was visually detected in the sEMG signal, typically characterized by low-frequency periodic components, an additional high-pass filter with a cutoff frequency of 60 Hz was applied to minimize this effect [1,48,49,50].

**Figure 4:**
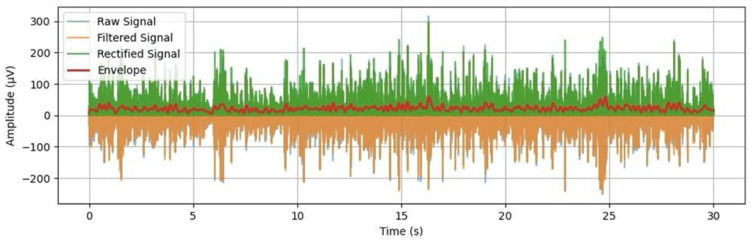
Representative signal processing obtained in animal SH13. Different stages of EMG signal treatment—in blue the raw signal, orange after filtering, green after rectification, and red the envelope obtained.

After filtering, the signals were full-wave rectified and smoothed using a 4th-order low-pass filter with a cutoff frequency of 5 Hz to generate the sEMG envelope [1,6]. This envelope reflects the amplitude modulation of muscle activation over time, providing an overall measure of the signal’s intensity/trend. From the envelope, descriptive statistics were extracted, including mean, standard deviation, and peak amplitude for each acquisition condition (Figure 4).

**Figure 5:**
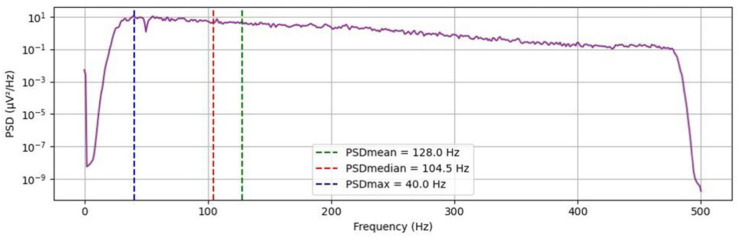
Representative signal obtained in animal SH13. After applying FFT algorithm to obtain frequency domain.

To further characterize the signals in the frequency domain, the Fast Fourier Transform (FFT) was applied to the filtered (non-rectified) data, as it can be seen in Figure 5 [6]. From the resulting power spectral density, which provides a statistical representation of a signal’s power distribution across different frequencies, the maximum (PSDmax), mean (PSDmean), and median (PSDmedian) frequencies were calculated, providing insight into the frequency decomposition of the sEMG signals and allowing comparison of noise profiles across electrode configurations.

In addition to the full frequency band (25–480 Hz), spectral analysis was also performed separately for two sub-bands: low-frequency (25–150 Hz) and high-frequency (150–300 Hz) [22,46]. The rationale for these frequency bands was derived from existing literature and the spectral regions most relevant for distinguishing signal and noise [22,46].

## 3. Results

Key metrics from both frequency and time domains were extracted for comparison between the dry and gel configurations during treadmill walking. This dynamic task allowed us to assess signal behavior under more realistic, movement-involved conditions. All computed metrics were compiled and organized for subsequent comparison and visualization of signal quality under the different hair conditions (with and without trichotomy); metrics were also stratified by electrode configuration and frequency band. A summary of the results is provided in Table 3.

**Figure 6:**
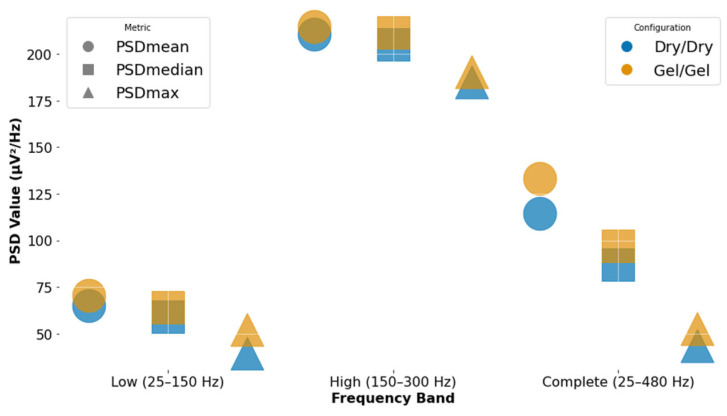
Illustration of frequency domain metrics divided by band and electrode configuration. Mean power spectral density represented by a circle (PSDmean), median power spectral density represented by a square (PSDmedian), and maximum power spectral density represented by a triangle (PSDmax). Soft polymeric dry electrode configuration is represented in blue color, and gel-based electrodes are represented by yellow color.

As expected in a dynamic task, greater variability was observed in amplitude-related features, particularly for the dry configuration. Full-band Power spectral density (PSD) values serve as a reference for overall spectral content during treadmill walking; Figure 6 shows the PSD metrics for each configuration across the three frequency bands. Both dry and gel electrodes yielded broadly comparable spectral features. However, the gel configuration showed slightly higher PSDmean, PSDmedian, and PSDmax values in all bands, particularly in the full-band (25–480 Hz), which may suggest more effective capture of neuromuscular activity with reduced spectral distortion. The dry setup exhibited slightly lower PSD values, which could reflect reduced sensitivity or increased noise filtering due to less optimal skin contact. Nonetheless, the consistency of the values across bands indicates that dry electrodes can still provide meaningful spectral information during dynamic conditions.

**Figure 7:**
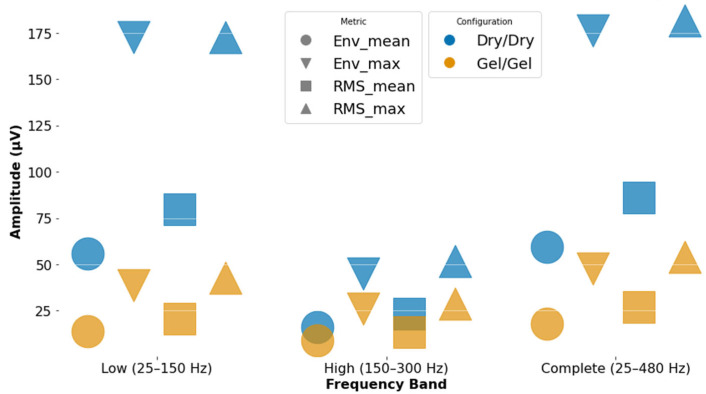
Illustration of temporal domain metrics results divided by band and electrode configuration. Mean envelope amplitude represented by a circle (env_mean), maximum envelope amplitude represented by a downward triangle (env_max), mean root mean square represented by a square (Mean RMS), and maximum root mean square represented by an upward triangle (Max RMS). Soft polymeric dry electrode configuration is represented in dark blue color, and gel-based electrodes are represented by yellow color.

Figure 7 summarizes the time-domain results. Here, the dry configuration yielded higher amplitude values across most metrics, notably in Max Env and Max RMS, which may indicate either stronger signal capture or increased susceptibility to motion artifacts caused by treadmill movement. In contrast, the gel electrodes produced lower but more stable amplitude values, especially in Mean Env and Mean RMS, suggesting improved signal stability under dynamic conditions. This trade-off between amplitude and stability should be considered when selecting electrodes for dynamic tasks.

## 4. Discussion

This work showed that soft polymeric dry electrodes can provide comparable signal quality to the one obtained with gelled electrodes in both static and dynamic conditions. The obtained results are consistent with previous research in veterinary sEMG, reporting the feasibility of dynamic muscle activity recording during treadmill locomotion [22,27]. However, by introducing dry electrodes into this context, the authors address an important gap identified in reviews such as Fuchs et al. (2022) [17], which highlighted the need for more practical, standardized, and less invasive approaches in clinical sEMG.

Hence, our results reinforce the feasibility of using dry electrodes for dynamic canine sEMG acquisition, although gelled electrodes may still offer slight advantages in terms of contact stability and signal clarity during movement. While gelled electrodes produced slightly higher spectral metrics, dry electrodes offer practical advantages, including reduced preparation time, greater animal tolerance, and environmental sustainability through reusability. In a study conducted by Steenbergen et al. (2023) [14], dry conductive elastomer (CE) electrodes also demonstrated performance comparable to traditional wet Ag/AgCl electrodes in human subjects, consistent with the results obtained in the present work. Unlike Ag/AgCl electrodes, which require gel and are limited by skin preparation, dermatological reactions, and short-term usability, CE electrodes offer flexibility, stretchability, and reduced mechanical mismatch without an electrolyte [14,51].

Electrochemical and functional sEMG testing showed that CE electrodes had lower impedance at relevant frequencies, which in this study translated into cleaner sEMG signals with reduced noise and motion artifacts. Classification using a backpropagation neural network yielded high accuracy (99.57% for CE-trained data and 94.30% for Ag/AgCl-trained data), demonstrating that CE electrodes can be effectively integrated into movement classification frameworks [14]. These findings support the use of solid, reusable CE electrodes as a viable long-term alternative to wet Ag/AgCl electrodes.

Our amplitude findings align with observations in human rehabilitation sEMG studies, where dry electrodes often yield higher peak values due to altered skin-electrode impedance and motion artifacts [7]. The slightly reduced PSD values did not compromise the extraction of key frequency metrics, in agreement with Suo et al. (2024) [6], who showed that spectral features can remain stable across electrode types if placement and fixation are consistent.

From a clinical perspective, the ability to obtain reliable sEMG data without trichotomy is particularly relevant for veterinary rehabilitation. Many canine patients, especially those undergoing long-term physiotherapy for IVDD, may require repeated sEMG assessment sessions; avoiding hair clipping can improve owner acceptance and reduce stress for the animal.

## 5. Conclusions

To our best knowledge, this study is the first to show that reusable soft polymeric dry electrodes can capture reliable paraspinal sEMG signals in dogs without hair clipping, offering comparable performance to gel-based electrodes. Their practicality, reusability, and animal-friendly application could expand the use of objective neuromuscular assessment in veterinary rehabilitation, especially for intervertebral disk disease. However, there are limitations to consider in future studies. Our study involved only one breed; despite reducing the number of potential confounding variables, it may limit generalizability to dogs with different coat types or body conformations. Future work should address this matter to permit the comparison between different breeds and myopathies, as it will permit a broader use of sEMG. In addition, the sample size was relatively small, and all subjects were healthy. While the assessment of animals with intervertebral disk disease was beyond the scope of the present study, future investigations will focus on this clinical population to further evaluate the applicability and generalizability of the findings. Long-term durability and repeated use performance of dry electrodes were not assessed. Future work should evaluate dry electrodes in multi-session rehabilitation monitoring, investigate optimal fixation techniques to minimize motion artifacts, and explore integration with wearable sEMG systems for remote physiotherapy monitoring. If validated in patient populations, this approach could expand the use of sEMG as an objective measure in veterinary rehabilitation, enabling more precise and individualized therapy adjustments. Considering the dynamic context of the text subjects (i.e., monitored during treadmill walking, and naturally excited and mobile), studying in greater detail the susceptibility of dry electrodes to noise and motion artifacts is of utmost importance.

## Figures and Tables

**Table 1:** Characteristics of the recruited dogs and inclusion or exclusion decision, * SH3 was excluded due to presence of pain at inicial clinical evaluation.

Animal	Age (Years)	Sex	Reproductive Status	Body Type	Coat Type	Weight (kg)	Body Condition Score (1 to 9)	Included/Excluded	Reason for Exclusion
SH1	2	male	intact	standard	long	7	4	included	
SH2	6	female	OVH	mini	smooth	6.8	6	included	
SH3 *	1	male	intact	standard	smooth	8.2	5	excluded	Pain at L4–L6
SH4	5	female	intact	mini	smooth	5	5	included	
SH5	2	male	intact	standard	smooth	11	5	included	
SH6	3	female	OVH	standard	wire	9	7	included	
SH7	3	male	intact	standard	smooth	6.7	4	included	
SH8	3	male	intact	standard	smooth	9	6	included	
SH9	2	male	spayed	standard	smooth	7.25	5	included	
SH10	5	male	intact	mini	wire	5.8	5	included	
SH11	5	male	spayed	Kanichen	smooth	3.5	4	included	
SH12	2	female	OVH	mini	smooth	4.5	4	included	
SH13	1	male	intact	standard	smooth	7.2	4	included	
Mean	3					7	5		
Standard deviation	1.7					2	1		
Median	3					7	5		

**Table 2:** Properties and application of the device and sensor, dry and wet electrodes used in the study.

Properties	Dry Electrode	Wet Electrode
Model and Manufacturer	SoftPulse^®^ Flex, Datwyler, Switzerland	Kendall™H124SG, UK
Diameter	13 mm	24 mm
Material	Dry conductive elastomer and contact area with an Ag/AgCl-based coating	Wet conductive and adhesive hydrogel with a polymeric Ag/AgCl coating
Inter-electrode Spacing	Fixed	Fixed
Inter-electrode Distance	approximately 2 cm	approximately 1 cm
Number of Electrodes	2	2
Recording Montage	Bipolar	Bipolar
Active vs. Passive Electrode	Passive	Passive
Grounding	Medial aspect of the tarsus	Medial aspect of the tarsus
Anatomical Location on the Muscle	0.5 cm lateral to the spinous processes of T12-L2 vertebra, overlying the *longissimus dorsi* muscle	0.5 cm lateral to the spinous processes of T12-L2 vertebra, overlying the *longissimus dorsi* muscle
Electrode Orientation	Parallel to muscle fibers	Parallel to muscle fibers

**Table 3:** EMG feature values for dry and gel electrodes, across low (25–150 Hz), complete (25–480 Hz), and high (150–300 Hz) frequency bands during treadmill walking. Features: Env Max—maximum envelope amplitude; Ret Max—time to maximum amplitude; Mean Ret—mean time to maximum amplitude; PSDmean, PSDmedian, PSDmax—mean, median, and maximum power spectral density; Mean RMS, Max RMS—mean and maximum root mean square.

Frequency Band	Configuration	Env Max	Ret Max	PSDmean	PSDmedian	PSDmax	Mean RMS	Max RMS
Low (25–150 Hz)	Dry	172.72	609.76	64.88	58.86	39.51	79.59	172.46
Low (25–150 Hz)	Gel	38.70	178.46	70.30	64.19	52.29	20.78	42.70
Complete (25–480 Hz)	Dry	176.37	792.33	114.26	87.18	43.59	85.85	181.87
Complete (25–480 Hz)	Gel	47.93	269.01	133.24	97.21	52.65	26.87	53.85
High (150–300 Hz)	Dry	44.86	285.22	210.43	205.17	185.1	23.51	51.77
High (150–300 Hz)	Gel	25.92	142.02	214.97	211.74	190.7	13.62	28.84

## Data Availability

The data presented in this study are available upon reasonable request made to the corresponding author.

## Abbreviations

The following abbreviations are used in this manuscript:

sEMG	Surface electromyography
ISNCSCI	International Standards for Neurological Classification of Spinal Cord Injury AIS—American Spinal Cord Association Impairment Scale
GRASSP	Graded redefined Assessment of strength, sensibility and Prehension
SCI	Spinal cord injury
CEDE	Consensus for Experimental Design in Electromyography
IVDD	Intervertebral disk disease
Ag/AgCl	Silver/silver chloride
FFT	Fast Fourier Transform
Hz	Hertz
Env	Envelope
RMS	Root mean square
RMSE	Root mean square error
PSD	Power spectral density
PSDmean	Mean power spectral density
PSDmax	Maximum power spectral density
PSDmedian	Median power spectral density
CE	Conductive elastomer

## References

[1] Merletti R., Muceli S. (2019). Tutorial. Surface EMG Detection in Space and Time: Best Practices. J. Electromyogr. Kinesiol..

[2] Meekins G.D., So Y., Quan D. (2008). American Association of Neuromuscular & Electrodiagnostic Medicine Evidenced-Based Review: Use of Surface Electromyography in the Diagnosis and Study of Neuromuscular Disorders. Muscle Nerve.

[3] Cram J.R. (2003). The History of Surface Electromyography. Appl. Psychophysiol. Biofeedback.

[4] Hermens H.J., Freriks B., Disselhorst-Klug C., Rau G. (2000). Development of Recommendations for SEMG Sensors and Sensor Placement Procedures. J. Electromyogr. Kinesiol..

[5] Cheng L., Li J., Guo A., Zhang J. (2023). Recent Advances in Flexible Noninvasive Electrodes for Surface Electromyography Acquisition. NPJ Flex. Electron..

[6] Suo M., Zhou L., Wang J., Huang H., Zhang J., Sun T., Liu X., Chen X., Song C., Li Z. (2024). The Application of Surface Electromyography Technology in Evaluating Paraspinal Muscle Function. Diagnostics.

[7] Al-Ayyad M., Owida H.A., De Fazio R., Al-Naami B., Visconti P. (2023). Electromyography Monitoring Systems in Rehabilitation: A Review of Clinical Applications, Wearable Devices and Signal Acquisition Methodologies. Electronics.

[8] Alcan V., Zinnuroğlu M. (2023). Current Developments in Surface Electromyography. Turk. J. Med. Sci..

[9] Besomi M., Hodges P.W., Van Dieën J., Carson R.G., Clancy E.A., Disselhorst-Klug C., Holobar A., Hug F., Kiernan M.C., Lowery M. (2019). Consensus for Experimental Design in Electromyography (CEDE) Project: Electrode Selection Matrix. J. Electromyogr. Kinesiol..

[10] Besomi M., Hodges P.W., Clancy E.A., Van Dieën J., Hug F., Lowery M., Merletti R., Søgaard K., Wrigley T., Besier T. (2020). Consensus for Experimental Design in Electromyography (CEDE) Project: Amplitude Normalization Matrix. J. Electromyogr. Kinesiol..

[11] Lathlean T.J.H., Ramachandran A.K., Sim S., Whittle I.R. (2024). The Clinical Utility and Reliability of Surface Electromyography in Individuals with Chronic Low Back Pain: A Systematic Review. J. Clin. Neurosci..

[12] Mitchell M.D., Yarossi M.B., Pierce D.N., Garbarini E.L., Forrest G.F. (2015). Reliability of Surface EMG as an Assessment Tool for Trunk Activity and Potential to Determine Neurorecovery in SCI. Spinal Cord.

[13] Balbinot G., Li G., Wiest M.J., Pakosh M., Furlan J.C., Kalsi-Ryan S., Zariffa J. (2021). Properties of the Surface Electromyogram Following Traumatic Spinal Cord Injury: A Scoping Review. J. Neuroeng. Rehabil..

[14] Steenbergen N., Busha I., Morgan A., Mattathil C., Levy Pinto A., Spyridakos F., Sokolovskiy I., Tahirbegi B., Chapman C., Cuttaz E. (2023). Surface Electromyography Using Dry Polymeric Electrodes. APL Bioeng..

[15] Li J., Wang P., Huang H.J. (2020). Dry Epidermal Electrodes Can Provide Long-Term High Fidelity Electromyography for Limited Dynamic Lower Limb Movements. Sensors.

[16] Valentin S., Zsoldos R.R. (2016). Surface Electromyography in Animal Biomechanics: A Systematic Review. J. Electromyogr. Kinesiol..

[17] Fuchs J., Bockay A., Liptak T., Ledecky V., Kuricova M. (2022). Practical Use of Electromyography in Veterinary Medicine—A Review. Vet. Med..

[18] Smit I.H., Parmentier J.I.M., Rovel T., van Dieen J., Serra Bragança F.M. (2024). Towards Standardisation of Surface Electromyography Measurements in the Horse: Bipolar Electrode Location. J. Electromyogr. Kinesiol..

[19] Zidan N., Sims C., Fenn J., Williams K., Griffith E., Early P.J., Mariani C.L., Munana K.R., Guevar J., Olby N.J. (2018). A Randomized, Blinded, Prospective Clinical Trial of Postoperative Rehabilitation in Dogs after Surgical Decompression of Acute Thoracolumbar Intervertebral Disc Herniation. J. Vet. Intern. Med..

[20] Martinez-Valdes E., Enoka R.M., Holobar A., McGill K., Farina D., Besomi M., Hug F., Falla D., Carson R.G., Clancy E.A. (2023). Consensus for Experimental Design in Electromyography (CEDE) Project: Single Motor Unit Matrix. J. Electromyogr. Kinesiol..

[21] Besomi M., Devecchi V., Falla D., McGill K., Kiernan M.C., Merletti R., van Dieën J.H., Tucker K., Clancy E.A., Søgaard K. (2024). Consensus for Experimental Design in Electromyography (CEDE) Project: Checklist for Reporting and Critically Appraising Studies Using EMG (CEDE-Check). J. Electromyogr. Kinesiol..

[22] Ribeiro A.M., Pereira D., Gaspar G.B., Dos Santos M.C., Plácido da Silva H., Requicha J.F. (2024). Surface Electromyography: A Pilot Study in Canine Spinal Muscles. MethodsX.

[23] Bockstahler B., Kräutler C., Holler P., Kotschwar A., Vobornik A., Peham C. (2012). Pelvic Limb Kinematics and Surface Electromyography of the Vastus Lateralis, Biceps Femoris, and Gluteus Medius Muscle in Dogs with Hip Osteoarthritis. Vet. Surg..

[24] Bockstahler B.B., Gesky R., Mueller M., Thalhammer J.G., Peham C., Podbregar I. (2009). Correlation of Surface Electromyography of the Vastus Lateralis Muscle in Dogs at a Walk with Joint Kinematics and Ground Reaction Forces. Vet. Surg..

[25] Negrão R.R., Rahal S.C., Kano W.T., Mesquita L.R., Hormaza J.M. (2022). Analysis of Time Series of Surface Electromyography and Accelerometry in Dogs. Biomed. Signal Process. Control..

[26] McLean H., Millis D., Levine D. (2019). Surface Electromyography of the Vastus Lateralis, Biceps Femoris, and Gluteus Medius in Dogs During Stance, Walking, Trotting, and Selected Therapeutic Exercises. Front. Vet. Sci..

[27] Miró F., Galisteo A.M., Garrido-Castro J.L., Vivo J. (2020). Surface Electromyography of the Longissimus and Gluteus Medius Muscles in Greyhounds Walking and Trotting on Ground Flat, Up, and Downhill. Animals.

[28] Bergknut N. (2011). Intervertebral Disc Degeneration in Dogs.

[29] Fenn J., Olby N.J. (2020). Canine Spinal Cord Injury Consortium (CANSORT-SCI) Classification of Intervertebral Disc Disease. Front. Vet. Sci..

[30] Abouzeid J., Grapes N., Khan S., De Decker S., Freeman P. (2025). Comparison of Clinical Features of Intervertebral Disc Extrusions in English Cocker Spaniels, French Bulldogs and Dachshunds. Animals.

[31] Schwartz J.A., Carrera-Justiz S., Repac J.A. (2024). Surface Electromyography of the Vastus Lateralis and Gluteus Medius Muscles in Post-Operative T3–L3 Hemilaminectomy Dogs: A Prospective Controlled Observational Study. Front. Vet. Sci..

[32] Millis D., Levine D. (2013). Canine Rehabilitation and Physical Therapy.

[33] Dewey C., da Costa R. (2015). Practical Guide to Canine and Feline Neurology.

[34] Radostits O.M., Mayhew I.G., Houston D.M. (2000). Veterinary Clinical Examination and Diagnosis.

[35] Pinello K., Geraz H., Salgueiro H., Cabral E., Vieira E., Mendonça M., Severo M., Ribeiro A.I., Ribeiro J. (2025). Socio-Geographic and Demographic Analysis of the Official National Registry Data of Dogs’ Population in Portugal in 2023. Data from SIAC. Vet. J..

[36] O’Neill D.G., McMillan K.M., Church D.B., Brodbelt D.C. (2023). Dog Breeds and Conformations in the UK in 2019: VetCompass Canine Demography and Some Consequent Welfare Implications. PLoS ONE.

[37] Bergknut N., Egenvall A., Hagman R., Gustås P., Hazewinkel H.A.W., Meij B.P., Lagerstedt A.-S. (2012). Incidence of Intervertebral Disk Degeneration–Related Diseases and Associated Mortality Rates in Dogs. J. Am. Vet. Med. Assoc..

[38] Brown E.A., Dickinson P.J., Mansour T., Sturges B.K., Aguilar M., Young A.E., Korff C., Lind J., Ettinger C.L., Varon S. (2017). FGF4 Retrogene on CFA12 Is Responsible for Chondrodystrophy and Intervertebral Disc Disease in Dogs. Proc. Natl. Acad. Sci. USA.

[39] Batcher K., Dickinson P., Giuffrida M., Sturges B., Vernau K., Knipe M., Rasouliha S.H., Drögemüller C., Leeb T., Maciejczyk K. (2019). Phenotypic Effects of FGF4 Retrogenes on Intervertebral Disc Disease in Dogs. Genes.

[40] Reunanen V.L.J., Jokinen T.S., Hytönen M.K., Junnila J.J.T., Lappalainen A.K. (2023). Evaluation of Intervertebral Disc Degeneration in Young Adult Asymptomatic Dachshunds with Magnetic Resonance Imaging and Radiography. Acta Vet. Scand..

[41] Packer R.M.A., Seath I.J., O’Neill D.G., De Decker S., Volk H.A. (2016). DachsLife 2015: An Investigation of Lifestyle Associations with the Risk of Intervertebral Disc Disease in Dachshunds. Canine Genet. Epidemiol..

[42] Martins Â., Gouveia D., Cardoso A., Carvalho C., Coelho T., Silva C., Viegas I., Gamboa Ó., Ferreira A. (2021). A Controlled Clinical Study of Intensive Neurorehabilitation in Post-Surgical Dogs with Severe Acute Intervertebral Disc Extrusion. Animals.

[43] Swallow J.S., Griffiths I.R. (1977). Age Related Changes in the Motor Nerve Conduction Velocity in Dogs. Res. Vet. Sci..

[44] Verga S.A., Pandeya S.R., Kowal J.B., Cochran R.J., Lim S., Sabol J.C., Coates J.R., Rutkove S.B. (2022). Electrical Impedance Myography in Healthy Dogs: Normative Values, Repeatability, and the Impact of Age. Front. Vet. Sci..

[45] Xu L., Peri E., Vullings R., Rabotti C., Van Dijk J.P., Mischi M. (2020). Comparative Review of the Algorithms for Removal of Electrocardiographic Interference from Trunk Electromyography. Sensors.

[46] Konrad P. (2005). The Abc of Emg.

[47] Galiana-Merino J.J., Ruiz-Fernandez D., Martinez-Espla J.J. (2013). Power Line Interference Filtering on Surface Electromyography Based on the Stationary Wavelet Packet Transform. Comput. Methods Programs Biomed..

[48] Strzecha K., Krakós M., Więcek B., Chudzik P., Tatar K., Lisowski G., Mosorov V., Sankowski D. (2021). Processing of EMG Signals with High Impact of Power Line and Cardiac Interferences. Appl. Sci..

[49] Boyer M., Bouyer L., Roy J.-S., Campeau-Lecours A. (2023). Reducing Noise, Artifacts and Interference in Single-Channel EMG Signals: A Review. Sensors.

[50] Clancy E.A., Morin E.L., Merletti R. (2002). Sampling, Noise-Reduction and Amplitude Estimation Issues in Surface Electromyography. J. Electromyogr. Kinesiol..

[51] Li G., Wang S., Duan Y.Y. (2018). Towards Conductive-Gel-Free Electrodes: Understanding the Wet Electrode, Semi-Dry Electrode and Dry Electrode-Skin Interface Impedance Using Electrochemical Impedance Spectroscopy Fitting. Sens. Actuators B Chem..

